# Integrated Analysis of DNA Methylation and Transcriptomic Dynamics in the Grape Variety ‘Cabernet Franc’ at Early and Late Stages of Fruit Development

**DOI:** 10.3390/plants15121815

**Published:** 2026-06-12

**Authors:** Qingtian Zhang, Shouming Shan, Xiaoyu Zhou, Pengfei Wang, Zhaobo Lang, Yujing Lin, Wei Ji, Ao Li

**Affiliations:** 1 Shandong Academy of Grape, Shandong Academy of Agricultural Sciences, Jinan 250100, China; tcszqt@163.com (Q.Z.); fengqiaoyouzi@126.com (P.W.); 2 College of Enology and Horticulture, Ningxia University, Yinchuan 750021, China; fxssm@163.com; 3 College of Biological Science and Technology, Jinan University, Jinan 250022, China; nivisz@163.com; 4 Institute of Advanced Biotechnology and School of Life Sciences, Southern University of Science and Technology, Shenzhen 518055, China; langzb@sustech.edu.cn (Z.L.); linyj3@sustech.edu.cn (Y.L.); 5 College of Horticulture, Shanxi Agricultural University, Jinzhong 030031, China; jiweiputao@163.com

**Keywords:** grapevine, DNA methylation, development process, gene expression

## Abstract

DNA methylation is a key epigenetic regulator in plant development. However, the changes in methylation patterns between the early and late stages of grape berry development, the two phases with the most pronounced morphological differences, and the respective roles of methylation at these stages remain largely unexplored. To investigate the dynamic DNA methylation changes during this stage and their regulatory role in fruit development, we constructed genome-wide methylation maps of grape at two key time points: the early development stage (7 days after flowering, 7DAF; hereafter referred to as S1) and the late development stages (78 days after flowering, 78DAF; hereafter referred to as S2). Global cytosine methylation increased from 12.57% (S1) to 14.16% (S2), driven primarily by a substantial increase in CHH methylation (from 5.88% to 7.92%; *p* < 0.001), whereas CG and CHG methylation showed no statistically significant change. Most differentially methylated regions (DMRs) were hypermethylated in S2, predominantly in the CHH context. Integrative methylome and transcriptome analysis revealed that CHH hypermethylation was associated with the downregulation of *YABBY5* (a berry size repressor) and upregulation of UGPase (a cell wall biosynthesis gene), suggesting a potential regulatory role in fruit expansion. Because our study compares only two time points, it cannot distinguish between gradual and stage-specific methylation changes, and functional validation of the identified genes is required. Nevertheless, these findings provides a valuable resource for understanding stage-specific DNA methylation dynamics and their association with gene expression during grape berry development.

## 1. Introduction

DNA methylation, a key epigenetic modification, is a critical regulator of gene expression during plant development and stress responses [1,2]. Dynamic changes in DNA methylation have been demonstrated to be essential for the development and ripening of various fruits [3,4,5,6]. For example, delayed fruit ripening and phenotypic variation in tomato are associated with DNA hypermethylation in the promoter region of the CNR (colorless non-ripening) gene, a master regulator of ripening [7]. Similar phenomena have been observed in other fruit species: in sweet orange, fruit ripening is characterized by global DNA hypermethylation, which is essential for normal ripening [5]; in strawberry, genome-wide methylation changes accompany the transition from unripe to ripe fruit [8]. Collectively, these findings demonstrate that dynamic DNA methylation is a critical and conserved regulatory mechanism controlling fruit development and ripening across diverse species.

Grape (*Vitis vinifera*) is an economically important fruit crop and a well-established model for studying non-climacteric fruit ripening. As such, understanding the epigenetic regulation of this process is of particular interest. DNA methylation, as a key epigenetic modification, plays a critical role in regulating gene expression and fruit quality formation during grape berry development [9]. Recent studies have begun to elucidate the epigenetic landscape underlying grape berry ripening. For instance, CHH hypermethylation was shown to contribute to early ripening in ‘Kyoho’ and its bud mutant [1], while dynamic methylation changes were observed during skin coloration in ‘Cabernet Franc’ [10]. Furthermore, genome-wide methylome analyses in multiple cultivars (‘Wink’, ‘Cabernet Sauvignon’, and ‘Fujiminori’) have consistently demonstrated that DNA hypermethylation, particularly in the CHH context, acts as a key epigenetic mechanism coordinating tissue-specific gene expression with metabolic shifts (e.g., anthocyanin biosynthesis and sugar metabolism) during berry ripening [6,11].

Despite these advances, existing studies have primarily focused on the middle and late stages of berry development, particularly around the veraison stage. DNA methylation patterns and their regulatory roles during the earlier stages of grape berry development (i.e., the fruit set stage, a key period marking the transition to fruit formation) have not yet been reported. Moreover, although the early and ripening stages differ markedly in fruit size, color, and metabolite content, it remains unclear whether these differences originate from changes in DNA methylation. Addressing this gap is essential for a comprehensive understanding of how epigenetic regulation shapes grape berry development from fruit set through maturation.

This study aims to address this knowledge gap by systematically analyzing the genome-wide DNA methylation dynamics of two previously uncompared critical stages of grape berry development: S1 and S2. By integrating methylome and transcriptome data, we aimed to identify stage-specific methylation changes and their potential associations with gene expression. Our findings provide foundational insights into the temporal dynamics of DNA methylation during grape berry development and generate testable hypotheses for future functional validation.

## 2. Results

### 2.1. Characteristics of the DNA Methylome in Grape (Cv. ‘Cabernet Franc’) Berry

**Figure 1:**
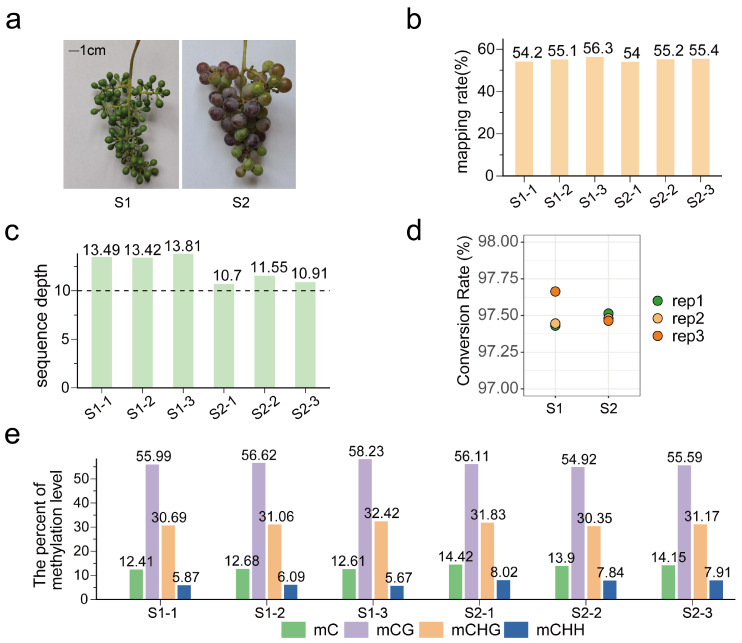
DNA methylation profiling of grape skin at different developmental stages. (**a**) Grape berry at various developmental stages. (**b**) Mapping rates of WGBS data. The percentage of clean reads that were uniquely mapped to the grape reference genome for each of the six libraries (S1-1, S1-2, S1-3, S2-1, S2-2 and S2-3). (**c**) Sequence depth for each replicate at the S1 and S2 stages. (**d**) Bisulfite conversion rates for each replicate at the S1 and S2 stages. (**e**) Percent of methylation levels (mCG, mCHG, mCHH) in grape skin at the S1 and S2 stages.

To investigate the DNA methylome in grape (cv. ‘Cabernet Franc’) berry, we utilized whole-genome bisulfite sequencing (WGBS) to generate single-base resolution methylation profiles of the grape skin at two developmental stages: S1 and S2. Samples were collected from both the S1 and S2, with three biological replicates per stage. Each sample generated at least 103,446,242 total reads (Table S1). The mapping rate and unique mapping rate for each sample exceeded 54% and 50.4%, respectively (Figure 1b). In each sequenced methylome, the average coverage per DNA strand was greater than 10-fold (Figure 1c). These sequencing coverage and depth metrics are consistent with previously published methylomes of Arabidopsis [12] and tomato [13]. The bisulfite conversion rate for each library was above 97.4% (Table S1 and Figure 1d), ensuring reliable methylation data.

Genome-wide DNA methylation analysis revealed distinct profiles in grape berry skin at the two developmental stages. The average cytosine methylation level was 12.57% in young berries (S1) and increased to 14.16% in ripening berries (S2) (Figure 1e). Context-specific analysis showed that, from the young to the ripening stage, CG and CHG methylation levels showed no statistically significant change (from 56.95% to 55.54%, *p* = 0.134; and from 31.39% to 31.12%, *p* = 0.7, respectively), whereas CHH methylation increased substantially (from 5.88% to 7.92%, *p* = 0.0001). Notably, the increase in global cytosine methylation was primarily driven by the gain in CHH context methylation, as the overall trend closely followed that of CHH.

### 2.2. Macro-Analysis of DNA Methylation Differences Between S1 and S2

**Figure 2:**
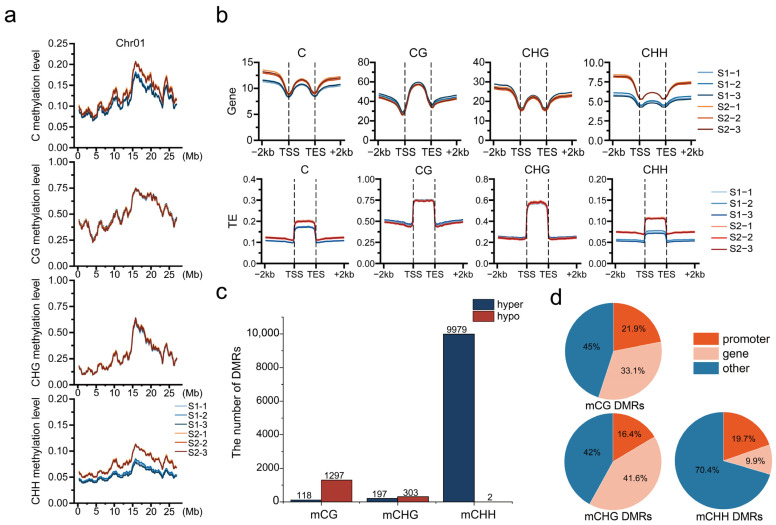
Distribution and characterization of DNA methylation and DMRs in grape skin at different developmental stages. (**a**) Methylation levels of CHH, CHG, CG, and overall cytosine contexts across chromosome 1 for grape skin at stages. (**b**) Distribution of DNA methylation across the gene body and TE regions at S1 (S1-1, S1-2 and S1-3) and S2 (S2-1, S2-2 and S2-3) stages. (**c**) The number of different DMRs in S2 vs. S1. (**d**) The proportion of DMRs in different genomic regions.

Context-specific analysis revealed that differences in DNA methylation between S1 and S2 were driven primarily by a substantial increase in CHH methylation. This trend is clearly illustrated on chromosome 1, where the rise in global methylation coincides with a sharp increase in CHH context methylation, while CG and CHG levels remained largely stable (Figure 2a). We next analyzed methylation dynamics specifically within gene and transposable element (TE) regions. Consistent with the genome-wide trend, CHH methylation increased in both gene and TE regions from S1 to S2 (Figure 2b). This increase was the primary driver of the overall elevation in DNA methylation within these genomic features. As expected, TEs were highly methylated in all sequence contexts. In genes, methylation levels were lower around the transcription start and end sites (TSS/TES), while the flanking upstream and downstream regions exhibited higher methylation than gene bodies (Figure 2b).

To further investigate DNA methylation dynamics during grape berry expansion, we identified DMRs between the early expansion stage and the ripening stage. A total of 11,896 DMRs were detected. Notably, hyper-DMRs (86.53%) vastly outnumbered hypo-DMRs (13.47%) (Figure 2c), indicating that an overall increase in DNA methylation occurs in S2 compared to S1. Notably, approximately 96.94% of hyper-DMRs occurred in the CHH context, highlighting CHH methylation as the primary form of change in S2. Analysis of their distribution by type revealed distinct patterns: among the 1415 CG-type DMRs, 468 were in gene regions and 310 in promoters; among the 500 CHG-type, 208 were in genes and 82 in promoters; and among the 9981 CHH-type, 1963 were in promoters and 991 in genes (Table S2). This indicates that CG- and CHG-type DMRs were more frequent in gene regions, while CHH-type DMRs predominated in promoters (Figure 2d).

### 2.3. Gene Ontology (GO) Enrichment Analysis of DMR-Related Genes

**Figure 3:**
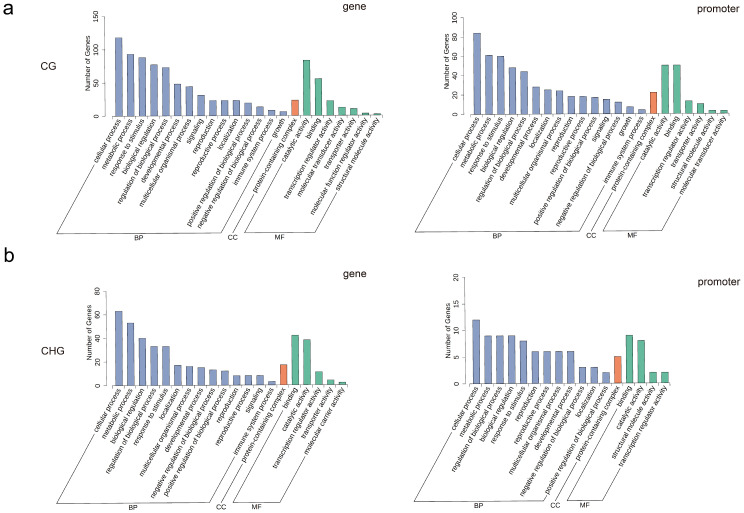
Gene ontology (GO) enrichment analysis of the genes related to the CG-type and CHG-type DMRs-related genes; BP: Biological Process (blue); CC: Cellular Component (orange); MF: Molecular Function (green). (**a**) Enriched GO terms of DMR overlapping gene body-related and promoter-related genes in the CG context. (**b**) Enriched GO terms of DMR overlapping gene body-related and promoter-related genes in the CHG context.

**Figure 4:**
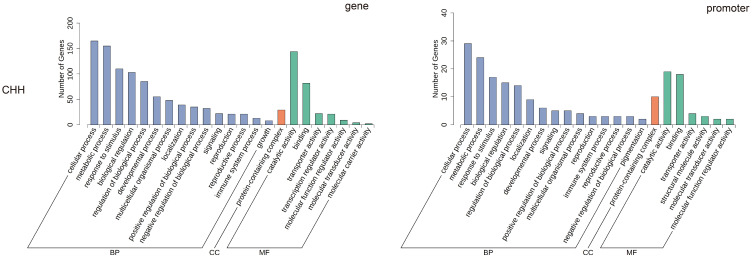
Enriched GO terms of DMR overlapping gene body-related and promoter-related genes in the CHH context; BP: Biological Process (blue); CC: Cellular Component (orange); MF: Molecular Function (green).

To explore the potential roles of these DMRs in S1 and S2, we performed Gene Ontology (GO) enrichment analysis. For CG-type DMRs overlapping with gene bodies, the most significantly enriched GO terms were “cellular response to light stimulus,” “cellular response to abiotic stimulus,” and “cellular polysaccharide metabolic process” (all with relatively smaller *p*-values; Figure 3a and Table S3). In contrast, for CG-type DMRs overlapping with promoters, the most significant enriched GO terms were “mRNA 3′-end processing,” “response to oxidative stress,” and “abscisic acid-activated signaling pathway” (Figure 3a and Table S4). For CHG-type DMRs overlapping with gene bodies, the most significantly enriched GO terms were “tRNA metabolic process,” “histidine metabolic process,” and “abscisic acid biosynthetic process” (all with relatively smaller *p*-values; Figure 3b and Table S3). In contrast, for CHG-type DMRs overlapping with promoters, the most significant enriched GO terms were “response to light intensity” “response to brassinosteroid,” and “integument development” (Figure 3b and Table S4). For CHH-type DMRs overlapping with gene bodies, the most significantly enriched GO terms were “cyanidin 3-O-glucoside biosynthetic process,” “regulation of photoreceptor cell differentiation,” and “glycosyl compound metabolic process” (all with relatively smaller *p*-values; Figure 4 and Table S3). In contrast, for CHH-type DMRs overlapping with promoters, the most significant enriched GO terms were “cyanidin 3-O-glucoside biosynthetic process” “response to iron ion starvation,” and “jasmonic acid and ethylene-dependent systemic resistance” (Figure 4 and Table S4).

### 2.4. RNA-Seq Analysis of S1 and S2

**Figure 5:**
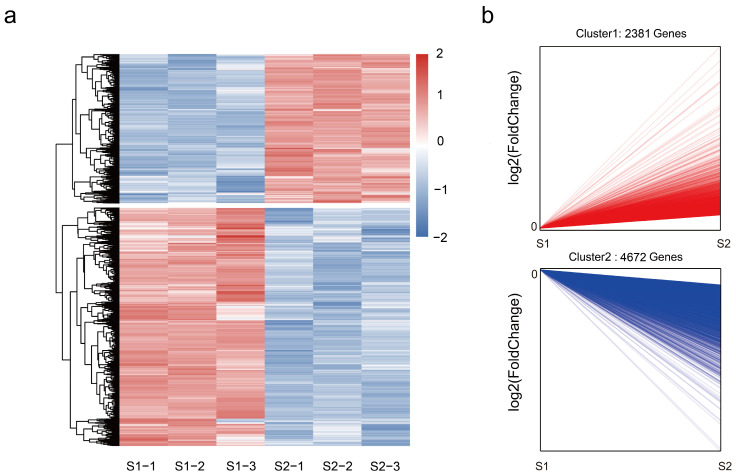
Transcriptomics analysis of grape skin at different developmental stages. (**a**) Heatmap showing the hierarchical clustering of DEGs between the S1 and S2 stages, with gene expression levels represented by color intensity (red for up-regulated and blue for down-regulated). (**b**) Clustering analysis of DEGs.

We performed RNA sequencing on grape skins at the S1 and S2 stages, with three biological replicates per stage. Each sample yielded at least 6 Gb of data. Analysis of expression levels revealed two distinct clusters based on expression patterns (Figure 5a). Compared to the S1, a total of 2381 genes (10.40% of total) were upregulated (Cluster 1) and 4672 genes (20.43% of total) were downregulated (Cluster 2) at the S2, indicating a significant shift in the global gene expression profile during berry expansion (Figure 5b). GO enrichment analysis revealed that the downregulated differentially expressed genes (DEGs)were significantly enriched in processes including photosynthesis, light harvesting, regulation of hormone levels, organic hydroxy compound metabolic processes, and UDP-glycosyltransferase activity (Figure S1). GO enrichment analysis indicated that the upregulated DEGs were primarily associated with responses to abscisic acid, antibiotics, and water, as well as ubiquitin-like protein transferase activity (Figure S2).

### 2.5. Correlation Between DNA Methylation and Gene Expression Between S1 and S2

**Figure 6:**
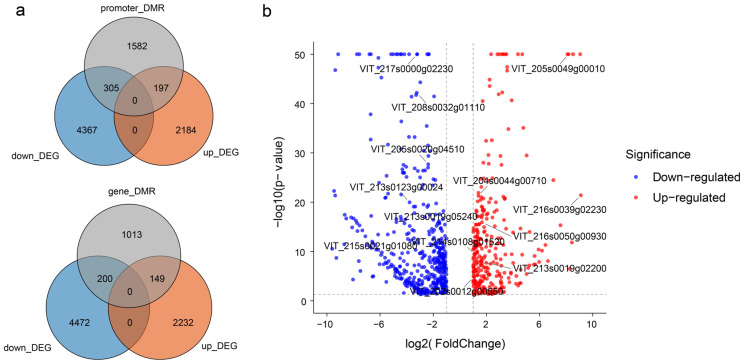
The DMRs-related DEGs. (**a**) Venn diagrams illustrating the DMR-related DEGs. (**b**) Volcano plot representing the *p*-value and log2 FC of DMR-related DEGs.

To investigate the relationship between DNA methylation and gene expression, we analyzed differentially expressed genes associated with DMRs in promoters or gene bodies (hereafter termed DMR-related DEGs). By integrating DMRs from gene body and promoter regions with DEGs via Venn diagram analysis, we found that, when comparing S2 to S1, 197 promoter DMR-related DEGs showed up-regulation and 305 showed down-regulation; for gene body DMR-related DEGs, 149 showed up-regulation and 200 showed down-regulation (Figure 6a). These 851 genes may be involved in the morphological differences between the two stages. A detailed classification of DMR-related DEGs by genomic region (promoter vs. gene body), methylation context (CG, CHG, CHH), methylation direction (hyper vs. hypo), and expression direction (up vs. down) is provided in Supplementary Data. To assess if their expression is associated with DNA methylation changes, we examined the transcriptomic data corresponding to these DMR-related genes (Figure 6b). This analysis revealed notable associations. GO enrichment analysis indicated that the DMR-related DEGs were primarily associated with response to water deprivation, pigment metabolic process and abscisic acid metabolic process (Figure S3). In the S2 vs. S1 comparison, increased gene body methylation was associated with up-regulated expression of several genes involved in distinct biological processes. Among these DMR-related DEGs, *YABBY5* (VIT_208s0032g01110) and *UGPase* (VIT_204s0044g00710) are particularly noteworthy. UGPase is a key enzyme involved in cell wall biosynthesis and fruit expansion [14], while *YABBY5* is a known determinant of berry size whose downregulation promotes fruit enlargement [15,16]. Other DMR-related DEGs, including *NAC078* [17], *IP5P2* [18], *CSLG2* [19], *GAUT14* [20,21], *UGT78D2* [22], a *PP2C* family member [18], *DTX35* [23], *TPS02* [24], *GME* [25,26], *TIFY6B* [25], and *CYP714A1* [27], are all associated with fruit development (Tables S5 and S6).

### 2.6. DNA Methylation and Gene Expression Changes in the RdDM Pathway

DNA methyltransferases and demethylases are crucial for maintaining DNA methylation homeostasis in plants [28,29]. In this study, we observed higher DNA methylation levels in grape skin after berry expansion. To determine whether this change correlates with altered expression of methylation-related enzymes, we analyzed the expression of ten DNA methyltransferase and three demethylase genes (Table S7) that had been previously identified in grape [6,12]. We found that, following grape development, the expression of *VvCMT1* (VIT_208s0007g06800), *VvCMT2a* (VIT_202s0033g00610), *VvCMT2b* (VIT_216s0039g02460), and *VvCMT3* (VIT_206s0004g01080) was significantly downregulated (Table S7). For the demethylase genes, the expression levels of *VvDML3* (VIT_206s0061g01270) were significantly upregulated, while the expression of *VvROS1* (VIT_208s0007g03920) and *VvDME* (VIT_213s0074g00450) was significantly downregulated (Table S7). Furthermore, the CHH context methylation levels in the gene body of *VvCMT2b* and the promoter region of *VvDNMT2* were significantly increased in the S2 compared to the S1 (Table S8). Notably, increased CHH methylation in the gene body of *VvCMT2b* was associated with its downregulated expression, whereas increased promoter CHH methylation of *VvDNMT2* was not accompanied by significant changes in its expression. These observations suggest that the relationship between CHH methylation changes and gene expression may be gene-specific and not directly causal.

## 3. Discussion

DNA methylation plays an important regulatory role in berry development, interacting with hormones and transcription factors [30]. Using WGBS, we analyzed the whole-genome DNA methylation of ‘Cabernet Franc’ at two previously uncompared critical developmental stages. The resulting coverage and sequencing depth were on par with published methylome data for Arabidopsis [12], orange [5], and tomato [13]. We observed a gradual increase in methylation levels with increasing distance from the transcription start and end sites across all sequence contexts in gene bodies and flanking regions (i.e., methylation levels were lowest near the TSS/TES and increased toward the gene body interior), consistent with findings in other grape cultivars [1,6]. Notably, prior studies across species such as strawberries [9], oranges [5], and tomatoes [13] have shown that both the dynamics of DNA methylation and the associated regulatory mechanisms vary significantly during fruit ripening. From S1 (7 DAF) to S2 (78 DAF) CG and CHG methylation levels in ‘Cabernet Franc’ showed no statistically significant change (from 56.95% to 55.54% and from 31.39% to 31.12%, respectively; *p* > 0.05), whereas CHH methylation increased significantly from 5.88% to 7.92% (*p* < 0.001; Figure 1e). Consistent with our findings, Shangguan et al. reported that from 40 DAF to 90 DAF after flowering, CG and CHG methylation levels in ‘Fujiminori’ decreased from 41.1% to 36.34% and from 21.35% to 19.6%, respectively, whereas CHH methylation increased from 2.96% to 4.74% [6]. Similarly, Li et al. observed that from 55 DAF to 77 DAF after flowering in ‘Cabernet Franc’, CG and CHG methylation levels decreased from 49.58% to 47.25% and from 29.65% to 28.26%, respectively, while CHH methylation increased from 5.39% to 5.85% [10]. The differences in absolute methylation levels between our study and Li et al. [10] likely reflect different developmental windows (broader coverage in our study: 7–78 DAF vs. 55–77 DAF) and environmental factors (year, location, climate). Despite these differences, both studies consistently show the same overall trend-stable or slightly decreasing CG/CHG and increasing CHH-supporting the CHH methylation may be involved in berry ripening. Therefore, a synthesis of previous and our studies indicates that the pattern of methylation changes during grape ripening is characterized by an overall decrease in CG and CHG methylation, accompanied by an overall increase in CHH methylation. Notably, the most pronounced increase occurred in the CHH context at the S2 stage (Figure 2c). Collectively, these results indicate that the genome-wide increase in DNA methylation was primarily driven by elevated CHH methylation, suggesting a potential regulatory role during this developmental transition [6].

To understand the enzymatic basis underlying these global methylation changes, we examined the expression of key DNA methyltransferases and demethylases. During the S1-to-S2 transition, the downregulation of demethylase genes (*VvROS1* and *VvDME*) suggests a coordinated shift toward hypermethylation. This coordinated shift coincides with the establishment of a stable methylation landscape during ripening and may be associated with the transcriptional reprogramming required for berry maturation. Reduced *VvROS1* expression accompanied increased methylation in its distal intergenic regions, implying autoregulatory repression-a pattern also observed in sweet orange [5]. Collectively, these changes are consistent with a decline in active demethylation alongside sustained or increased maintenance methylation, which may contribute to the net increase in DNA methylation.

To explore the functional relevance of these methylation changes, we performed Gene Ontology (GO) enrichment analysis on genes associated with differentially methylated regions (DMRs). Genes associated with DMRs were significantly enriched in processes such as mRNA 3′-end processing and response to light intensity. Given that mRNA 3′-end formation is a critical regulatory point in eukaryotic gene expression [31], we hypothesize that this process may affect fruit development, potentially through associations with gene expression. Furthermore, DMR-related genes overlapping with DEGs were implicated in abscisic acid metabolism and hormone level regulation. Together with the enrichment in mRNA processing, these findings suggest that DMR-associated genes may function as upstream regulators of development-related genes. Notably, the term ‘cyanidin 3-O-glucoside biosynthetic process’ was significantly enriched in both gene body-associated and promoter-associated DMRs within the CHH context. This repeated enrichment suggests that CHH methylation may be specifically associated with anthocyanin metabolism. This observation is consistent with the berry coloring phenotype observed at the S2 stage (ripening), during which anthocyanins accumulate substantially in the skin. Whether CHH methylation directly regulates the expression of anthocyanin biosynthesis genes or merely reflects the transcriptional activation of this pathway, however, remains to be determined through functional validation.

In this study, we observed a complex, gene-specific relationship between DNA methylation and gene expression in grapevine when comparing S2 and S1 developmental stages (Table S5). Both promoter and gene body methylation were associated with either increased or decreased expression. This complexity is illustrated across multiple pathways. In ABA (Abscisic acid) signaling, *IP5P2* (VIT_202s0012g00550) and *PP2CA* (VIT_213s0019g02200) were both upregulated—*IP5P2* with increased gene body methylation, and *PP2CA* with promoter methylation changes. Since ABA inhibits early fruit development [18], their upregulation may alleviate this inhibition. Supporting this, *CYP714A1*, a GA (Gibberellin)-inactivating enzyme [27], was downregulated with increased gene body methylation, potentially maintaining active GA levels to promote cell elongation [22]. For developmental regulation, *YABBY5* and *UGPase* stand out as potential key regulators of berry morphology. The downregulation of *YABBY5* at S2, associated with increased promoter CHH methylation, is consistent with its proposed role as a negative regulator of fruit size [22,23]. This methylation-associated repression may relieve growth inhibition, thereby allowing berry expansion. Conversely, the upregulation of *UGPase* at S2, associated with increased gene body methylation, may enhance cell wall biosynthesis and fruit expansion [14]. These opposing regulatory patterns—repression of a growth inhibitor and activation of a biosynthetic enzyme—may act synergistically to promote fruit enlargement during the S1-to-S2 transition. However, the precise molecular mechanisms by which CHH methylation affects the expression of these genes require further investigation. These results indicate that the relationship between gene body methylation and gene expression is not unidirectional: increased gene body methylation was associated with upregulation in some genes (e.g., *UGPase*) but with downregulation in others (e.g., *TIFY6B*). This bidirectional relationship may depend on gene function, sequence context, or interactions with other epigenetic modifications. The two-stage design captures only the start and end points of berry development and cannot distinguish whether methylation changes occur gradually or at specific transition points. More importantly, both DMR-DEG overlap analysis and any potential correlation analysis provide only associative, not causal, evidence. While our study identifies candidate genes whose methylation and expression patterns co-vary, determining whether these changes directly regulate transcription, result from transcriptional activity, or reflect independent biological processes requires functional validation (e.g., targeted methylation editing or genetic perturbation). Taken together, these results suggest that stage-specific methylation changes are closely associated with fruit development, potentially accompanying the activation of biosynthetic pathways (flavonoid, cell wall), repression of growth inhibitors (e.g., *YABBY5*), and fine-tuning of ABA, GA, and JA signaling. However, whether these methylation changes directly drive or merely reflect the developmental transition warrants further investigation.

## 4. Materials and Methods

### 4.1. Plant Materials

This study used *V. vinifera* (cv. ‘Cabernet Franc’) as the study species. Grape skin tissues were collected at two developmental stages: S1 and S2. For each stage, three biological replicates were independently collected and prepared. Immediately after collection, the samples were frozen in liquid nitrogen and stored at −80 °C until subsequent genomic DNA and total RNA extraction.

### 4.2. Whole-Genome Bisulfite Sequencing and Analysis

Genomic DNA was extracted from grape skin tissues using the DNeasy Plant Maxi Kit (Qiagen, Hilden, Germany). DNA purity and concentration were assessed using a NanoPhotometer (Implen GmbH, Munich, Germany) and Qubit 2.0 Fluorometer (Life Technologies, Carlsbad, CA, USA), respectively. DNA was fragmented to ~250 bp using a Bioruptor sonication system, followed by end-repair, dA-tailing, adapter ligation, and size selection via agarose gel electrophoresis. Bisulfite conversion was performed using the EZ DNA Methylation-Gold Kit (Zymo Research Corp., Irvine, CA, USA), converting unmethylated cytosines to uracil while leaving methylated cytosines unchanged. Libraries were PCR-amplified, purified, and sequenced on an Illumina platform. Raw reads were trimmed with Trimmomatic to remove adapters and low-quality reads (unknown bases > 10% or bases with Q < 20 > 10%). The *V. vinifera* genome and TE annotations were retrieved from NCBI (https://www.ncbi.nlm.nih.gov/datasets/genome/GCF_030704535.1/, accessed on 11 June 2025). Clean reads were mapped to the reference genome using BSMAP [32], duplicate reads were removed, and methylation ratios were calculated using the methratio.py script. Genome-wide methylation levels for CG, CHG, and CHH contexts were calculated as weighted methylation: Σ(methylated reads across all analyzed cytosines)/Σ(total coverage reads across all analyzed cytosines). Average coverage depths for S1 and S2 were 13× and 11×, respectively, exceeding the 10× threshold required for reliable methylation estimates [33]. Regions 2 kb upstream/downstream of the transcription start site (TSS) and transcription termination site (TTS), as well as gene body regions (TSS to TTS), were extracted. Each upstream/downstream region was divided into 20 bins, and each gene body into 40 bins.

DMRs were identified for each sequence context using the methylKit R package (version 4.4.2). For each stage, three biological replicates were analyzed independently. For each replicate, methylation calls were extracted at individual CG sites. A methylRawList object was then created containing all six samples (three from S1 and three from S2). Differentially methylated cytosines (DMCs) were identified by comparing S2 vs. S1 using Fisher’s exact test with BH correction (*p* ≤ 0.05). DMCs within 200 bp were merged into candidate regions. A candidate region was defined as a DMR if it met three criteria: (i) ≥5 DMCs; (ii) average methylation difference (|S2 − S1|) ≥ 0.1 (10%); and (iii) combined Fisher’s exact test *p* ≤ 0.05 [34]. DMRs were classified as CG-, CHG-, or CHH-DMR based on sequence context, and were then overlapped with gene annotations to identify DMR-associated genes.

### 4.3. Gene Ontology (GO) Analysis

Based on the identified DMR-associated genes, we performed Gene Ontology (GO) enrichment analysis using the online cloud platform Metware Cloud (https://cloud.metware.cn, accessed on 11 November 2025) [35]. The enrichment analysis was conducted using a hypergeometric statistical test. The background gene set comprised all protein-coding genes annotated in the *V. vinifera* reference genome (GCF_030704535.1). Transposable element (TE) regions in the grape genome were annotated using RepeatMasker (version 4.1.0) with the *V. vinifera*-specific repeat library obtained from Repbase. The resulting TE annotation file was used for subsequent methylation analysis. GO terms with a Benjamini–Hochberg false discovery rate (FDR) adjusted *p*-value ≤ 0.05 were considered significantly enriched. For each significant GO term, we reported the observed gene count, expected gene count, fold enrichment, and adjusted *p*-value.

### 4.4. RNA Extraction, Sequencing, and Transcriptome Analysis

Total RNA was extracted from grape skin tissues at the S1 and S2 developmental stages, with three biological replicates per stage, to construct RNA-seq libraries. The libraries were sequenced on an Illumina NovaSeq 4000 platform, generating 150 bp paired-end reads. Raw RNA sequencing data were processed with Trimmomatic to trim adapters and remove low-quality reads [36]. Clean reads were then aligned to the grape reference genome using HISAT2 (version 2.0.4) software [37]. Transcript assembly was performed with StringTie (version 1.2.3) [38], and gene expression levels were quantified as FPKM (fragments per kilobase of transcript per million mapped reads) using StringTie. For cross-sample comparisons, FPKM values were converted to TPM (transcripts per million) using the formula: TPM = (FPKM/ΣFPKM) × 1,000,000. TPM values were used for all visualizations (PCA, heatmaps, clustering) to ensure optimal cross-sample comparability. Differential expression analysis was performed using the DESeq2 R package. Genes with fewer than 10 reads across all samples were filtered out prior to analysis. The DESeq2 model was fitted using the negative binomial distribution, and DEGs between S2 and S1 were identified using the Wald test. The resulting *p*-values were adjusted using the Benjamini–Hochberg (BH) method, and a false discovery rate (FDR, *p*adj) ≤ 0.05 together with |log_2_ fold change| > 1 was considered statistically significant. K-cluster analysis of the DEGs was performed using the OmicShare tool (http://www.omicshare.com/tools, accessed on 10 January 2026). Gene expression values were normalized using Z-score transformation (mean = 0, SD = 1). Euclidean distance was used as the distance metric, and the number of clusters (K) was set to 2 based on the elbow method.

## Data Availability

The WGBS and RNA-seq datasets used in this study have been deposited in the NCBI database under the BioProject ID PRJNA1470057. Further inquiries can be directed to the corresponding author.

## Supplementary Materials

The following supporting information can be downloaded at: https://www.mdpi.com/article/10.3390/plants15121815/s1. Figure S1: Enriched GO terms of downregulated DEGs; Figure S2: Enriched GO terms of upregulated DEGs; Figure S3: Enriched GO terms of DMR-related DEGs; Table S1:Mapping statistics of bisulfite sequencing; Table S2: The number of differentially methylated regions (DMRs); Table S3: The enriched GO terms of the differentially methylated regions (DMRs) overlapping gene body-related genes during skin fruit development of grape; Table S4: The enriched GO terms of the differentially methylated regions (DMRs) overlapping promoter regions-related genes during skin fruit development of grape; Table S5: Expression of the genes involved in grape fruit development; Table S6: DNA methylation of the genes involved in grape fruit development; Table S7: Expression of genes involved in DNA methylation and demethylation during fruit development of grape; Table S8: DNA methylation of genes related to DNA methylation and demethylation title; Supplementary Data: A detailed classification of DMR-related DEGs by genomic region (promoter vs. gene body), methylation context (CG, CHG, CHH), methylation direction (hyper vs. hypo), and expression direction (up vs. down).

## Abbreviations

The following abbreviations are used in this manuscript:

DAF	days after flowering
DMRs	differentially methylated regions
WGBS	whole-genome bisulfite sequencing
DEGs	differentially expressed genes
CNR	colorless non-ripening
