# Supplementary material for: Integrated Analysis of DNA Methylation and Transcriptomic Dynamics in the Grape Variety ‘Cabernet Franc’ at Early and Late Stages of Fruit Development
Source: Plants (Basel). 2026 Jun 12;15(12):1815. doi: 10.3390/plants15121815 (PMC13307283; doi:10.3390/plants15121815)
Supplement: Supplementary file 1 [file plants-15-01815-s001.zip › supplementary.pdf]

Supplementary Figure

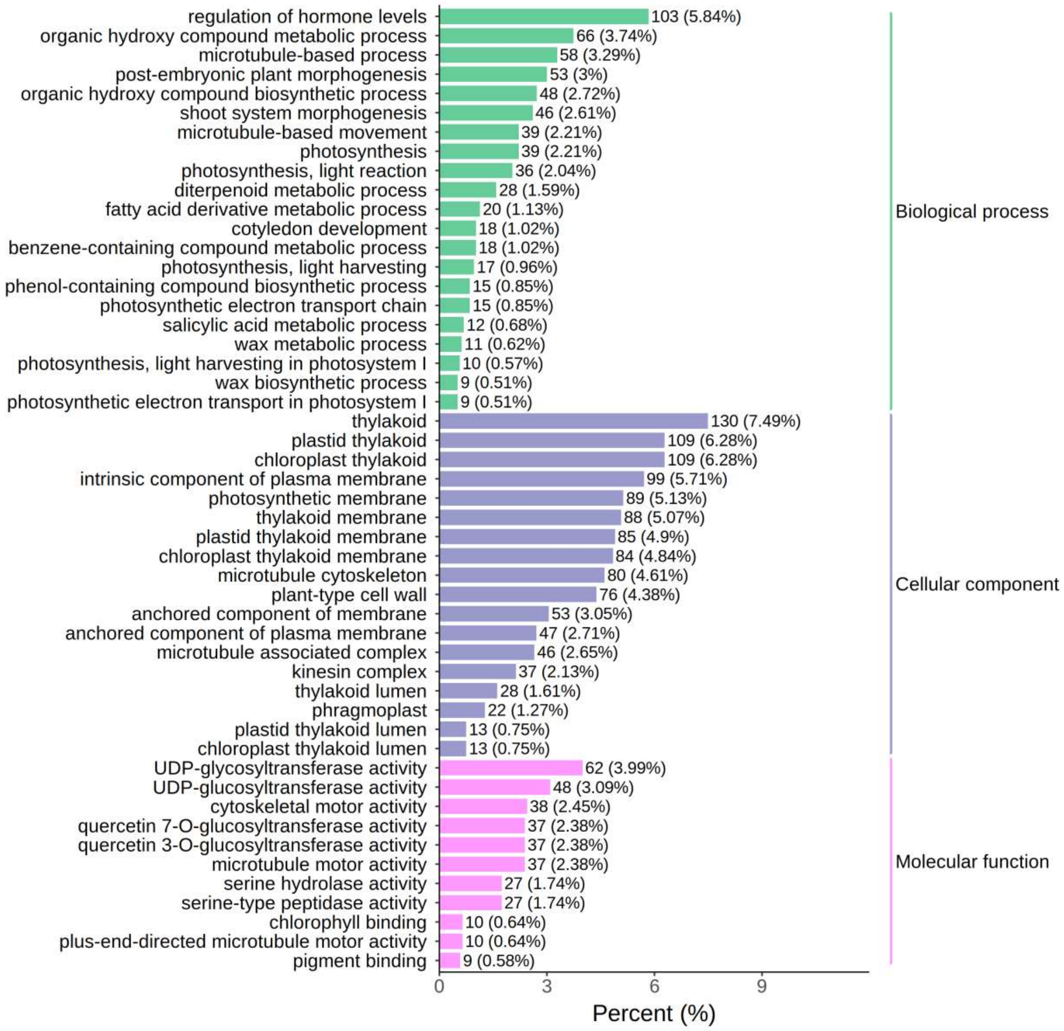

Figure S1 Enriched GO terms of downregulated DEGs.

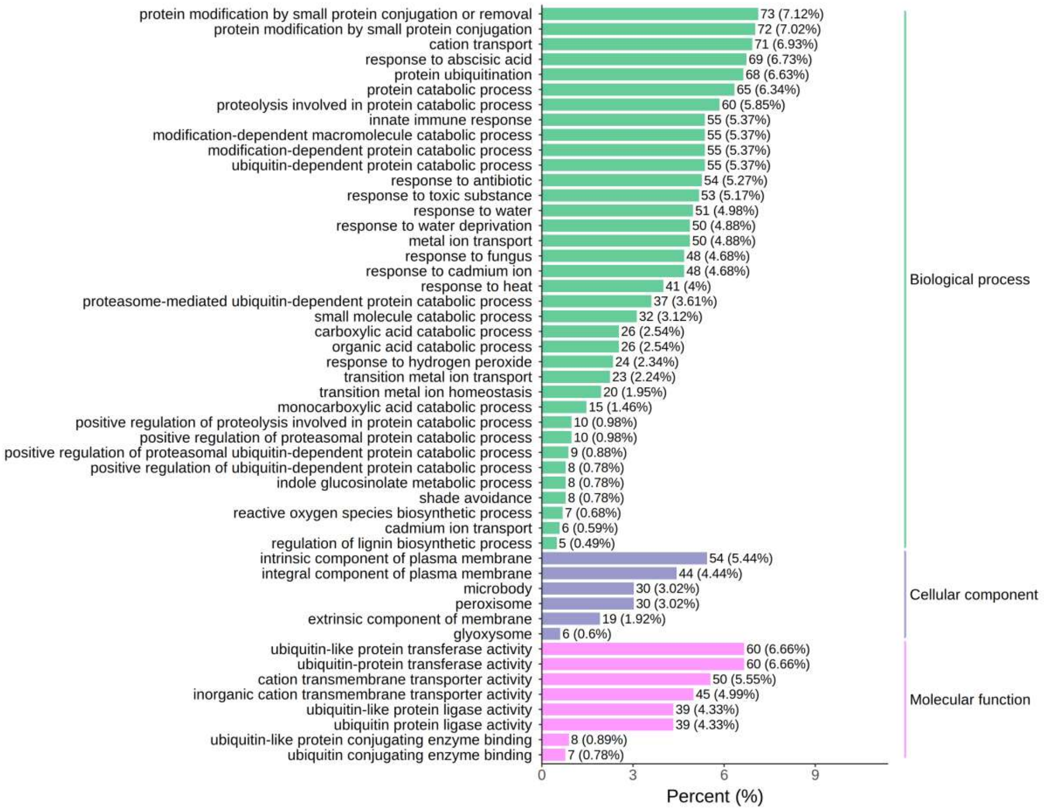

Figure S2 Enriched GO terms of upregulated DEGs.

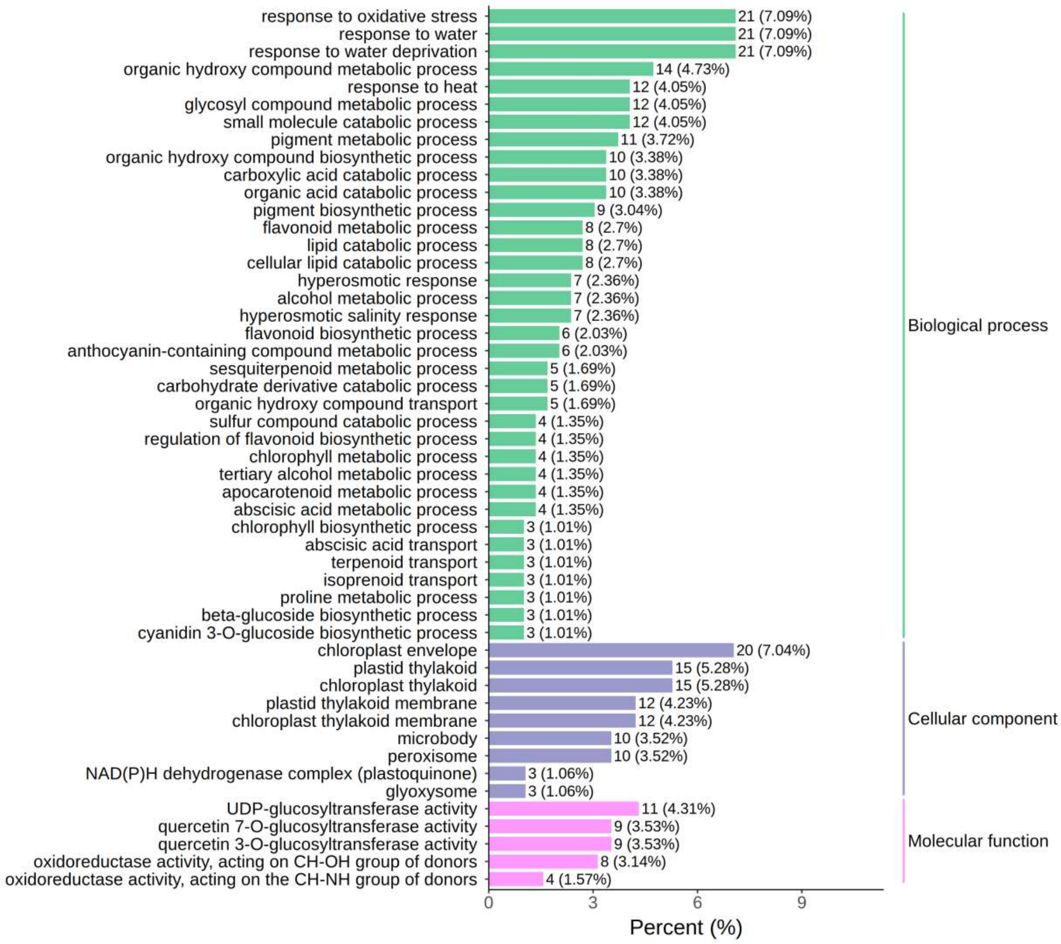

Figure S3 Enriched GO terms of DMR-related DEGs.

Supplementary Tables

Table S1 Mapping statistics of bisulfite sequencing

sample	total reads	aligned pairs	unique pairs
S1-1	115826795	62801794(54.2%)	58366053(50.4%)
S1-2	110541208	60913298(55.1%)	56632681(51.2%)
S1-3	111963674	63068663(56.3%)	58328653(52.1%)
S2-1	111037557	60004290(54.0%)	55970459(50.4%)
S2-2	107539091	59313254(55.2%)	55178019(51.3%)
S2-3	103446242	57281843(55.4%)	53155560(51.4%)

Table S2 The number of differentially methylated regions (DMRs)

	CG	CHG	CHH	total
genic region	468	208	991	1,667
promoter region	310	82	1963	2,355
all	1,415	500	9,981	11,896

Table S3 The enriched GO terms of the differentially methylated regions (DMRs) overlapping gene body-related genes during skin fruit development of grape

	Description	*P*-value
CG	cellular response to light stimulus	1.96773E-05
	cellular response to radiation	4.8306E-05
	red, far-red light phototransduction	0.000145026
	red or far-red light signaling pathway	0.000166866
	cellular response to red or far red light	0.000189281
	phototransduction	0.000214281
	cellular carbohydrate metabolic process	0.00025535
	detection of light stimulus	0.000256608
	cellular response to abiotic stimulus	0.000322891
	cellular response to environmental stimulus	0.000322891
	detection of external stimulus	0.001820284
	detection of abiotic stimulus	0.001820284
	cellular polysaccharide metabolic process	0.002135123
	response to red or far red light	0.002170703
	cellular polysaccharide catabolic process	0.002418307
	root system development	0.003007846
	regulation of meristem growth	0.003139265
	response to far red light	0.003693841
	oligosaccharide metabolic process	0.004261408
	photomorphogenesis	0.004311392
	carbohydrate metabolic process	0.004742237
CHG	tRNA metabolic process	0.000893686
	negative regulation of transcription by RNA polymerase II	0.001221408
	tRNA aminoacylation for protein translation	0.001298056
	amino acid activation	0.001461338
	tRNA aminoacylation	0.001461338
	histidine metabolic process	0.002530315
	imidazole-containing compound metabolic process	0.002530315
	positive regulation of cell differentiation	0.002622174
	P-body assembly	0.003077178
	abscisic acid biosynthetic process	0.00575842
	apocarotenoid biosynthetic process	0.00575842
	tertiary alcohol biosynthetic process	0.00575842
	cellular amino acid metabolic process	0.007024989
	pseudouridine synthesis	0.007384523
	chromatin organization	0.009456662
	negative regulation of RNA metabolic process	0.011148459
	abscisic acid metabolic process	0.01333346

	apocarotenoid metabolic process	0.01333346
	tertiary alcohol metabolic process	0.01333346
	positive regulation of cell development	0.01447357
	histone modification	0.015480691
	regulation of cell differentiation	0.015480691
	DNA conformation change	0.017833874
	negative regulation of RNA biosynthetic process	0.018861481
	negative regulation of nucleic acid-templated transcription	0.018861481
	negative regulation of nucleobase-containing compound metabolic process	0.019884843
	secretion by cell	0.027176869
	peptide biosynthetic process	0.027527824
	DNA metabolic process	0.028195628
	secretion	0.032767891
	negative regulation of gene expression	0.034505072
	vascular transport	0.036082889
	phloem transport	0.036082889
	negative regulation of nitrogen compound metabolic process	0.038186664
	sesquiterpenoid biosynthetic process	0.043115907
	negative regulation of DNA-templated transcription	0.044677616
	regulated exocytosis	0.044948047
	alcohol biosynthetic process	0.044948047
	regulation of membrane potential	0.046808649
	amide biosynthetic process	0.049076552
CHH	cyanidin 3-O-glucoside biosynthetic process	0.000104375
	beta-glucoside biosynthetic process	0.000104375
	regulation of photoreceptor cell differentiation	0.000143795
	regulation of animal organ morphogenesis	0.000317748
	positive regulation of cell death	0.000341613
	glycosyl compound metabolic process	0.000505063
	columnar/cuboidal epithelial cell differentiation	0.000904433
	negative regulation of apoptotic signaling pathway	0.000904433
	S-glycoside catabolic process	0.001101156
	glycosinolate catabolic process	0.001101156
	glucosinolate catabolic process	0.001101156
	regulation of neuron differentiation	0.001105988
	axon guidance	0.001224939
	regulation of R7 cell differentiation	0.001224939
	cyanidin 3-O-glucoside metabolic process	0.001300445
	beta-glucoside metabolic process	0.001300445
	glycoside biosynthetic process	0.001392756
	regulation of secondary metabolite biosynthetic process	0.001523629
	regulation of neurogenesis	0.001730903

regulation of protein ubiquitination	0.002350935
regulation of secondary metabolic process	0.002582311
neuron projection guidance	0.003180163
regulation of protein modification process	0.003298588
regulation of nervous system development	0.003395074
S-glycoside metabolic process	0.003435895
glycosinolate metabolic process	0.003435895
glucosinolate metabolic process	0.003435895
glycosyl compound catabolic process	0.003444409
regulation of protein modification by small protein conjugation or removal	0.003874417
axonogenesis	0.004612009
glycoside metabolic process	0.005135028
regulation of catalytic activity	0.006115299
compound eye development	0.00637786
axon development	0.00637786
sulfur compound catabolic process	0.006573506
chemotaxis	0.006921328
regulation of endopeptidase activity	0.008674744
monocarboxylic acid metabolic process	0.009244159
monocarboxylic acid biosynthetic process	0.009361017
taxis	0.009713939
response to hypoxia	0.010127133
negative regulation of catalytic activity	0.010473643
sucrose metabolic process	0.012362451
pyridine-containing compound metabolic process	0.01238915
neuron development	0.013070647
carbohydrate metabolic process	0.013137688
negative regulation of protein modification process	0.013197937
regulation of cysteine-type endopeptidase activity involved in apoptotic process	0.013840472
cell morphogenesis involved in neuron differentiation	0.013840472
neuron projection morphogenesis	0.013840472
cell projection morphogenesis	0.013840472
plasma membrane bounded cell projection morphogenesis	0.013840472
regulation of apoptotic signaling pathway	0.013840472
regulation of peptidase activity	0.015162724
response to organic cyclic compound	0.015301291
epithelial cell differentiation	0.015414601
secondary metabolic process	0.016088914
negative regulation of protein localization	0.017085495
response to decreased oxygen levels	0.017936676
regulation of anatomical structure morphogenesis	0.018547173

regulation of hydrolase activity	0.01855267
pigmentation	0.018853642
anthocyanin-containing compound biosynthetic process	0.019951682
positive regulation of programmed cell death	0.019951682
compound eye photoreceptor cell differentiation	0.020187442
regulation of protein targeting	0.020187442
oligosaccharide metabolic process	0.020376158
cell part morphogenesis	0.020719369
response to oxygen levels	0.02126476
carbohydrate derivative catabolic process	0.021278496
negative regulation of molecular function	0.02180632
neuron differentiation	0.022658189
negative regulation of transferase activity	0.022682849
negative regulation of protein metabolic process	0.023214428
eye photoreceptor cell differentiation	0.023903176
negative regulation of Wnt signaling pathway	0.023903176
negative regulation of cysteine-type endopeptidase activity involved in apoptotic process	0.023903176
negative regulation of canonical Wnt signaling pathway	0.023903176
positive regulation of intrinsic apoptotic signaling pathway	0.023903176
apoptotic process	0.024091213
generation of neurons	0.025982713
carbohydrate catabolic process	0.026271461
negative regulation of protein phosphorylation	0.026903044
positive regulation of apoptotic process	0.026903044
hyperosmotic response	0.027174079
carbohydrate derivative biosynthetic process	0.027292312
regulation of system process	0.027874645
photoreceptor cell differentiation	0.027874645
regulation of gene silencing by RNA	0.027874645
regulation of cell development	0.031055196
negative regulation of phosphorylation	0.03151285
regulation of cellular protein metabolic process	0.031881854
proline metabolic process	0.032089965
xylan catabolic process	0.032089965
detoxification of nitrogen compound	0.032089965
nervous system development	0.032947997
regulation of cysteine-type endopeptidase activity	0.033962884
nicotinamide nucleotide metabolic process	0.035267728
compound eye morphogenesis	0.036537627
cyanide metabolic process	0.036537627
defense response to Gram-negative bacterium	0.036537627
flavone biosynthetic process	0.036537627

regulation of morphogenesis of an epithelium	0.036537627
positive regulation of apoptotic signaling pathway	0.036537627
neurogenesis	0.037396496
pyridine-containing compound biosynthetic process	0.037515266
regulation of hormone levels	0.0378122
pyridine nucleotide metabolic process	0.038263097
neuron projection development	0.039150287
flavone metabolic process	0.041206485
regulation of intrinsic apoptotic signaling pathway	0.041206485
organic acid biosynthetic process	0.042842883
carboxylic acid biosynthetic process	0.042842883
regulation of cell death	0.043316934
hyperosmotic salinity response	0.043460797
anthocyanin-containing compound metabolic process	0.043460797
regulation of mitochondrion organization	0.046085749
positive regulation of endopeptidase activity	0.046085749
regulation of canonical Wnt signaling pathway	0.046085749
cell fate commitment	0.047702084
phospholipid biosynthetic process	0.048160705
glycosyl compound biosynthetic process	0.049944346

Table S4 The enriched GO terms of the differentially methylated regions (DMRs) overlapping promoter regions-related genes during skin fruit development of grape

	Description	*P*-value
CG	mRNA 3'-end processing	0.000752075
	RNA 3'-end processing	0.003731446
	response to oxidative stress	0.003832568
	abscisic acid-activated signaling pathway	0.004536715
	centromere complex assembly	0.006259648
	long-day photoperiodism, flowering	0.007349153
	purine nucleobase transmembrane transport	0.007349153
	response to abscisic acid	0.008394035
	purine nucleobase transport	0.008517713
	nucleobase transport	0.009763648
	peptide catabolic process	0.009763648
	long-day photoperiodism	0.009763648
	cellular response to abscisic acid stimulus	0.011505154
	cellular response to alcohol	0.013704789
	purine-containing compound transmembrane transport	0.013949268
	regulation of locomotion	0.017181509
	protein modification by small protein removal	0.017181509
	miRNA processing	0.020515723
	negative regulation of transcription by RNA polymerase II	0.023699612
	protein transmembrane transport	0.025775322
	mRNA cleavage	0.026126503
	regulation of vesicle fusion	0.026126503
	embryo development ending in seed dormancy	0.027386361
	photoperiodism	0.02906644
	positive regulation of chromatin organization	0.030171862
	regulation of chromatin organization	0.03137847
	protein targeting to mitochondrion	0.0322818
	positive regulation of RNA metabolic process	0.03361551
	protein localization to mitochondrion	0.034448098
	establishment of protein localization to mitochondrion	0.034448098
	root hair cell development	0.035022388
	protein glycosylation	0.03756811
	macromolecule glycosylation	0.03756811
	protein-DNA complex assembly	0.038875662
	regulation of response to external stimulus	0.040694594
	protein transmembrane import into intracellular organelle	0.04365052

	vegetative to reproductive phase transition of meristem	0.043694614
	positive regulation of DNA-templated transcription	0.045564165
	cellular response to acid chemical	0.045564165
	mRNA polyadenylation	0.046079022
	regulation of ethylene-activated signaling pathway	0.046079022
	miRNA-mediated gene silencing	0.046079022
	regulation of phosphorelay signal transduction system	0.046079022
	RNA polyadenylation	0.048556189
	glycoprotein biosynthetic process	0.048666848
	regulation of organelle organization	0.051828472
	response to antibiotic	0.053202733
	positive regulation of gene expression	0.056251765
	calcium ion transmembrane transport	0.05632922
	positive regulation of RNA biosynthetic process	0.057115709
	positive regulation of nucleic acid-templated transcription	0.057115709
	mRNA processing	0.057748719
	protein deubiquitination	0.058927528
CHG	response to light intensity	0.001914797
	response to high light intensity	0.005739426
	response to brassinosteroid	0.014344622
	photoinhibition	0.017759158
	negative regulation of photosynthesis, light reaction	0.017759158
	integument development	0.017759158
	flower development	0.0194027
	pollen tube reception	0.019518501
	regulation of ER to Golgi vesicle-mediated transport	0.019518501
	negative regulation of photosynthesis	0.019518501
	reproductive shoot system development	0.021839248
	purine nucleobase transmembrane transport	0.023028208
	purine nucleobase transport	0.024778583
	nucleobase transport	0.026525979
	positive regulation of transcription elongation by RNA polymerase II	0.026525979
	photosystem II repair	0.0282704
	regulation of anthocyanin biosynthetic process	0.0282704
	positive regulation of DNA-templated transcription, elongation	0.0282704
	gonad development	0.030011851
	isopentenyl diphosphate biosynthetic process	0.030011851
	protein secretion	0.030011851
	protein repair	0.030011851

	development of primary sexual characteristics	0.030011851
	isopentenyl diphosphate metabolic process	0.030011851
	pyrimidine-containing compound catabolic process	0.030011851
	peptide secretion	0.031750336
	phosphate ion transport	0.031750336
	purine-containing compound transmembrane transport	0.031750336
	pollination	0.033066055
	regulation of transcription elongation by RNA polymerase II	0.033485862
	pyrimidine ribonucleoside metabolic process	0.033485862
	pyrimidine nucleoside metabolic process	0.035218432
	multi-multicellular organism process	0.035746544
	regulation of DNA-templated transcription elongation	0.038674724
	regulation of photosynthesis, light reaction	0.038674724
	histone H3-K4 methylation	0.038674724
	response to organic cyclic compound	0.039882545
	salicylic acid mediated signaling pathway	0.042119251
	negative regulation of cell growth	0.043837115
	regulation of defense response to virus	0.043837115
	floral whorl development	0.043852476
	regulation of anthocyanin metabolic process	0.045552052
CHH	cyanidin 3-O-glucoside biosynthetic process	0.000903364
	beta-glucoside biosynthetic process	0.000903364
	response to iron ion starvation	0.00156127
	ribosomal small subunit assembly	0.001752563
	cyanidin 3-O-glucoside metabolic process	0.003120005
	beta-glucoside metabolic process	0.003120005
	SRP-dependent cotranslational protein targeting to membrane	0.003657793
	cotranslational protein targeting to membrane	0.003941741
	pigmentation	0.00423563
	jasmonic acid and ethylene-dependent systemic resistance	0.004852961
	protein targeting to ER	0.007316741
	establishment of protein localization to endoplasmic reticulum	0.007316741
	glycoside biosynthetic process	0.008929932
	protein targeting to membrane	0.009790812
	protein localization to endoplasmic reticulum	0.010687193
	regulation of actin filament polymerization	0.011148536
	regulation of innate immune response	0.011935673

regulation of actin polymerization or depolymerization	0.012097214
regulation of actin filament length	0.012097214
regulation of protein polymerization	0.013080126
anthocyanin-containing compound biosynthetic process	0.013584265
DNA-templated transcription initiation	0.014096776
glycoside metabolic process	0.015683933
intracellular protein transmembrane transport	0.015683933
establishment of protein localization to membrane	0.015683933
regulation of actin filament organization	0.016782782
RNA phosphodiester bond hydrolysis, endonucleolytic	0.01734425
protein transmembrane transport	0.02026964
regulation of supramolecular fiber organization	0.02026964
anthocyanin-containing compound metabolic process	0.021493794
protein targeting	0.021498061
regulation of actin cytoskeleton organization	0.023386432
ribosomal small subunit biogenesis	0.023386432
regulation of immune response	0.023517782
regulation of actin filament-based process	0.024685133
ribosome assembly	0.025345396
flavonoid biosynthetic process	0.027369171
regulation of cytoskeleton organization	0.033071487
protein localization to membrane	0.03381456
DNA-templated transcription	0.03426883
nucleic acid-templated transcription	0.034692993
pattern recognition receptor signaling pathway	0.037739623
regulation of polysaccharide biosynthetic process	0.037739623
RNA biosynthetic process	0.03999687
DNA damage response, signal transduction by p53 class mediator	0.041100855
mitotic G1 DNA damage checkpoint signaling	0.041100855
mitotic G1/S transition checkpoint signaling	0.041100855
intrinsic apoptotic signaling pathway	0.041100855
auxin-activated signaling pathway	0.041597187
regulation of cellular component size	0.043227841
positive regulation of hydrolase activity	0.043227841
transcription by RNA polymerase I	0.044450646
protein import into mitochondrial matrix	0.044450646
very long-chain fatty acid biosynthetic process	0.044450646
glycosyl compound biosynthetic process	0.04571828
regulation of immune system process	0.047175815
regulation of polysaccharide metabolic process	0.047789034

	positive regulation of cysteine-type endopeptidase activity involved in apoptotic process	0.047789034
	transcription preinitiation complex assembly	0.047789034

Table S5 Expression of the genes involved in grape fruit development

Gene ID	S1-1 TPM	S1-2 TPM	S1-3 TPM	S2-1 TPM	S2-2 TPM	S2-3 TPM	*p*-value
VIT_204s0044g00710 (UGPase)	398.21	342.45	385.41	740.16	861.11	891.8805	6.78E-20
VIT_214s0108g01520 (GAUT14)	37.98	40.47	39.17	64.93	87.35	63.41	7.9E-10
VIT_208s0032g01110 (YABBY5)	110.15	133.55	171.67	10.53	13.37	13.07	3.233E-43
VIT_213s0019g05240 (NAC078)	131.75	131.74	127.93	241.65	293.88	235.10	1.47E-19
VIT_216s0039g02230 (UGT78D2)	1.17	0.12	0.20	423.42	194.50	160.07	2.377E-33
VIT_202s0012g00550 (IP5P2)	57.10	51.24	78.61	92.83	137.14	100.35	1.68E-05
VIT_213s0019g02200 (PP2CA)	181.48	193.49	332.26	717.24	603.10	507.03	7.91E-09
VIT_216s0050g00930 (DTX35)	25.97	32.55	36.75	75.59	90.59	89.06	5.12E-17
VIT_205s0049g00010 (CSLG2)	0	0.12	0.40	68.44	60.73	57.35	8.23E-73
VIT_213s0123g00024 (TPS02)	48.81	55.36	55.09	1.72	2.02	5.80	1.28E-36
VIT_205s0020g04510 (GME)	1328.75	1182.00	1555.82	253.65	173.22	229.11	4.41E-28
VIT_217s0000g02230 (JAZ3)	179.85	194.01	238.53	17.42	20.20	17.86	5.75E-69
VIT_215s0021g01080 (CYP714A1)	2.54	3.35	2.68	0	0	0	2.03E-09

Table S6 DNA methylation of the genes involved in grape fruit development

Gene ID	S1 vs S2							
	C		CG		CHG		CHH	
	gene boby	promoter	gene boby	promoter	gene boby	promoter	gene boby	promoter
VIT_204s0044g00710 (UGPase)	up	-	up	-	-	-	-	-
VIT_214s0108g01520 (GAUT14)	-	up	-	-	-	-	-	-
VIT_208s0032g01110 (YABBY5)	-	up	-	-	-	-	-	up
VIT_213s0019g05240 (NAC078)	up	-	-	-	-	-	-	-
VIT_216s0039g02230 (UGT78D2)	-	up	-	-	-	-	-	-
VIT_202s0012g00550 (IP5P2)	up	-	-	-	-	-	-	-
VIT_213s0019g02200 (PP2CA)	-	up	-	-	-	-	-	-
VIT_216s0050g00930 (DTX35)	-	up	-	-	-	-	-	up
VIT_205s0049g00010 (CSLG2)	up	-	-	-	-	-	-	-
VIT_213s0123g00024 (TPS02)	-	up	-	-	-	-	-	up
VIT_205s0020g04510 (GME)	-	up	-	-	-	-	-	-
VIT_217s0000g02230 (JAZ3)	up	-	-	-	-	-	up	-
VIT_215s0021g01080 (CYP714A1)	up	-	-	-	-	-	-	-

Table S7 Expression of genes involved in DNA methylation and demethylation during fruit development of grape

Gene ID	Annotation	S1 TPM	S2 TPM	S1 vs S2 $\log_2$FoldChange	S1 vs S2 *P*_value
VIT_207s0130g00390	*VvMET2a*	45.61432817	41.53670517	-	-
VIT_207s0130g00380	*VvMET2b*	9.322798827	12.67842438	-	-
VIT_212s0035g01770	*VvMET3*	0.262920563	0.822109001	-	-
VIT_214s0066g01040	*VvDRM1*	30.55119251	12.88095623	-1.053563401	1.92e-07
VIT_205s0020g00450	*VvDRM2*	55.99830095	30.29083011	-	-
VIT_208s0007g06800	*VvCMT1*	6.053388784	1.23132857	-1.834955463	3.45e-06
VIT_202s0033g00610	*VvCMT2a*	0.219197023	0	-4.809037503	0.02185
VIT_216s0039g02460	*VvCMT2b*	2.808619072	0.088826892	-4.590371497	2.95e-08
VIT_206s0004g01080	*VvCMT3*	3.295002043	0.324047804	-1.980720366	0.0017
VIT_204s0008g05060	*VvDNMT2*	20.06135985	19.47692881	-	-
VIT_208s0007g03920	*VvROS1*	128.4776211	43.76947677	-1.239769689	2.11e-08
VIT_213s0074g00450	*VvDME*	90.06186542	23.21305167	-1.704271437	2.02e-11
VIT_206s0061g01270	*VvDML3*	0	0.869624875	7.48868731191189	4.90e-08

Table S8 DNA methylation of genes related to DNA methylation and demethylation

Gene ID	Annotation	S1 vs S2					
		CG		CHG		CHH	
		gene boby	promoter	gene boby	promoter	gene boby	promoter
VIT_207s0130g00390	*VvMET2a*	-	-	-	-	-	-
VIT_207s0130g00380	*VvMET2b*	-	-	-	-	-	-
VIT_212s0035g01770	*VvMET3*	-	-	-	-	-	-
VIT_214s0066g01040	*VvDRM1*	-	-	-	-	-	-
VIT_205s0020g00450	*VvDRM2*	-	-	-	-	-	-
VIT_208s0007g06800	*VvCMT1*	-	-	-	-	-	-
VIT_202s0033g00610	*VvCMT2a*	-	-	-	-	-	-
VIT_216s0039g02460	*VvCMT2b*	-	-	-	-	up	-
VIT_206s0004g01080	*VvCMT3*	-	-	-	-	-	-
VIT_204s0008g05060	*VvDNMT2*	-	-	-	-	-	up
VIT_208s0007g03920	*VvROS1*	-	-	-	-	-	-
VIT_213s0074g00450	*VvDME*	-	-	-	-	-	-
VIT_206s0061g01270	*VvDML3*	-	-	-	-	-	-
